# A Unified Linear Viscoelastic Model of the Cell Nucleus Defines the Mechanical Contributions of Lamins and Chromatin

**DOI:** 10.1002/advs.201901222

**Published:** 2020-03-05

**Authors:** Oren Wintner, Nivi Hirsch‐Attas, Miriam Schlossberg, Fani Brofman, Roy Friedman, Meital Kupervaser, Danny Kitsberg, Amnon Buxboim

**Affiliations:** ^1^ Department of Cell and Developmental Biology The Alexander Silberman Institute of Life Sciences The Hebrew University of Jerusalem Jerusalem 9190401 Israel; ^2^ Alexander Grass Center for Bioengineering The Rachel and Selim Benin School of Computer Science and Engineering Jerusalem 9190416 Israel; ^3^ The de Botton Institute for Protein Profiling The Nancy and Stephen Grand Israel National Center for Personalized Medicine Weizmann Institute of Science Rehovot 7610001 Israel

**Keywords:** nuclear lamins, nucleus mechanics, nucleus mechanobiology

## Abstract

The cell nucleus is constantly subjected to externally applied forces. During metazoan evolution, the nucleus has been optimized to allow physical deformability while protecting the genome under load. Aberrant nucleus mechanics can alter cell migration across narrow spaces in cancer metastasis and immune response and disrupt nucleus mechanosensitivity. Uncovering the mechanical roles of lamins and chromatin is imperative for understanding the implications of physiological forces on cells and nuclei. Lamin‐knockout and ‐rescue fibroblasts and probed nucleus response to physiologically relevant stresses are generated. A minimal viscoelastic model is presented that captures dynamic resistance across different cell types, lamin composition, phosphorylation states, and chromatin condensation. The model is conserved at low and high loading and is validated by micropipette aspiration and nanoindentation rheology. A time scale emerges that separates between dominantly elastic and dominantly viscous regimes. While lamin‐A and lamin‐B1 contribute to nucleus stiffness, viscosity is specified mostly by lamin‐A. Elastic and viscous association of lamin‐B1 and lamin‐A is supported by transcriptional and proteomic profiling analyses. Chromatin decondensation quantified by electron microscopy softens the nucleus unless lamin‐A is expressed. A mechanical framework is provided for assessing nucleus response to applied forces in health and disease.

## Graphical abstract

The cell nucleus is evolutionarily‐optimized to deform while protecting the genome under load. A viscoelastic model of nucleus resistance to physiologically relevant stresses that is conserved across cell types and nucleoskeletal perturbations is presented. An inherent timescale separates between elastic stretching set by lamin‐A and viscoelastic fluid deformation. Both A‐ and B‐type lamins contribute to steady‐state stiffness whereas lamin‐A and chromatin decondensation increase viscosity.

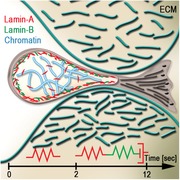

## 1 Introduction

The nucleus is the largest organelle in the cell where the genome is transcribed, replicated, sequence‐repaired, and protected from externally applied and cell‐generated forces. Owing to its size, the nucleus impedes cell migration across narrow interstitial spaces.^[^ 1, 2, 3 ^]^ Excessive shear stresses applied on the nucleus by stiff extracellular boundaries combined with nucleus softening can lead to nuclear envelope (NE) herniation and rupture, exchange of nucleoplasmic and cytoplasmic contents and squeezing out DNA repair components that increases the incidence of double‐strand DNA breaks and chromosomal aberrations.^[^ 4, 5, 6, 7 ^]^ Nucleus mechanics is dominated by the thin filamentous networks of A‐ and B‐type lamins that line the inner side of the inner nuclear membrane (INM),^[^ 8, 9 ^]^ and chromatin.^[^ 10, 11 ^]^ In addition to their structural functions, lamins play important regulatory roles in cellular differentiation,^[^ 12, 13, 14 ^]^ embryonic development,^[^ 15 ^]^ 3D genome organization,^[^ 16 ^]^ nuclear mechanotransduction^[^ 17 ^]^ gene expression,^[^ 18 ^]^ and DNA replication.^[^ 19 ^]^ Forces can be directly converted into biochemical cues at the NE via tension‐dependent modulation of lamin‐A phosphorylation (tension‐suppressed)^[^ 20 ^]^ and emerin phosphorylation (tension‐enhanced),^[^ 21 ^]^ which associates with lamin‐A/C. Consistently, genetic disorders originating from mutations in lamin genes or other genes encoding proteins of the nuclear lamina (termed laminopathies) exhibit aberrant nuclear mechanics that are linked with pathology.^[^ 22, 23, 24 ^]^ Hence, uncovering the mechanical roles of A‐ and B‐type lamins and chromatin will broaden our mechanistic understanding of biological processes that involve physical deformation of the nucleus in health and disease.

Nucleus rheology depends on the time scales and length scales of induced deformations as characterized via various methods, including substrate stretching,^[^ 25 ^]^ indentation,^[^ 26, 27 ^]^ microneedle‐based micromanipulation,^[^ 28, 29 ^]^ and micropipette aspiration.^[^ 30, 31 ^]^ This complex viscoelastic response reflects the broad range of weak and strong interactions between lamins and chromatin^[^ 32 ^]^ both at the NE^[^ 10, 11 ^]^ and at the nucleoplasm (NP) and to the stabilization of condensed chromatin structures within lamin‐associated domains (LADs).^[^ 33 ^]^ Micromanipulation measurements revealed a typical length scale of deformations (≈3 µm), below which nucleus elastic resistance is dominated by chromatin whereas resistance to large deformations is dominated by lamin‐A/C.^[^ 29, 30 ^]^ Unlike isolated chromatin fibers that stretch elastically,^[^ 34 ^]^ the rheological properties of nuclear chromatin ranges between dominantly elastic to dominantly viscous and modulated by NE tethers.^[^ 35 ^]^ Lamin‐A/C levels, more so than lamin‐B1, vary across tissue‐resident cell types to scale with tissue microelasticity, thus tuning nucleus stiffness to extracellular stiffness.^[^ 14 ^]^ Consistent with the thin and low‐density meshwork of soft lamin filaments,^[^ 9 ^]^ the lamina itself (both A‐ and B‐type lamin meshworks) renders little mechanical strength to the nucleus.^[^ 36, 37 ^]^

Using micropipette aspiration, we measured the mechanical response of nuclei within intact cells over physiological length scales and stresses. To evaluate the viscoelastic contributions of lamin‐A alone, lamin‐B1 alone, lamin‐A and ‐B1 together and lamin‐A phosphorylation states, we established stable cultures of lamin‐knockout and lamin‐rescue mouse embryonic fibroblastic cells. The mechanical roles of chromatin were analyzed using a pharmaceutical inhibitor of chromatin deacetylation that reversibly induces chromatin decondensation. The integrated mechanical roles of lamins and chromatin were analyzed with reference to RNA and protein profiles and transmission electron microscopy. We provide for the first time a minimal linear viscoelastic model that probes the interrelated mechanical contributions of lamins and chromatin. A time scale is emerging (≈2 s), which separates between two temporal regimes, exhibiting distinctive nuclear mechanical responses to applied stress. At short times, the nucleus stretches elastically and becomes effectively softer at long times. Effective nucleus stiffness is dominated by lamins A and B1 and condensed chromatin. In cells that express lamin‐A, chromatin decondensation stiffens the nucleus potentially due to newly generated NE interactions. At steady state, the nucleus deforms viscously as controlled exclusively by lamin‐A. We demonstrate the generality of our viscoelastic model not only across lamin expression profiles, phosphorylation states and chromatin condensation states, it is also validated for nuclei of embryonic and pluripotent stem cells whose lamin‐A and B1 levels and chromatin compaction is much lower compared with fibroblastic cells.

## 2 Results

### 2.1 Generating Immortalized Lamin‐Null Mouse Embryonic Fibroblast Cell Lines

Triple knockout (TKO) mouse embryonic fibroblast (MEF) cells that do not express Lmna, Lmnb1 and Lmnb2 were isolated from E13.5 sacrificed embryos following injection of TKO mouse embryonic stem cells into mouse blastocysts (kindly contributed by the Zheng lab; Experimental Section).^[^ 15, 38 ^]^ Lamin‐rescued MEFs were generated by lentiviral transfection of TKO cells. To isolate transfected cells and control lamin expression levels, we designed lentiviral constructs that encode lamin‐A and lamin‐B1 that are 3'‐conjugated to mCherry (Lamin‐A) or venus (Lamin‐B1) fluorescent reporter proteins under a doxycycline‐inducible promoter. The fluorescent reporter sequences were fused via Thosea asigna virus 2A (T2A) self‐cleaving peptide. In this manner, potential mechanical contributions due to aggregation or ectopic nuclear localization of the conjugated fluorescent proteins were avoided. In this manner, A‐rescue, B1‐rescue and AB1‐rescue MEFs were generated by transfecting TKO cells with lamin‐A, lamin‐B1, and lamin‐A together with lamin‐B1, respectively (Figure S1a, Supporting Information). Lamin‐A was rescued to ≈50% WT level in A‐ and AB1‐rescue nuclei. To study the mechanical contributions of Serine‐22 (S22) phosphorylated lamin‐A, which accounts for 5% to 10% of total lamin‐A in interphase cells,^[^ 20 ^]^ we designed and transfected TKO cells with permanently phosphorylated (S22D) and nonphosphorylatalbe (S22A) lamin‐A mimetic constructs. S22 phosphorylation by Cdk1 and its homologous site on B‐type lamins is the primary trigger for disassembly of the filamentous lamin network predominantly during cell cycle,^[^ 39, 40, 41 ^]^ although S22 phosphorylation is also mechanically regulated in interphase cells by applied nuclear tension.^[^ 20 ^]^ As a control, *Lmna* knockout MEF cell line (AKO) was also included in addition to WT, TKO, and lamin‐rescue cells.^[^ 42 ^]^

Protein levels of lamins were independently evaluated via quantitative immunofluorescence (*n* > 30 per condition, IF; Figure S1b‐i, Supporting Information), immunoblotting (WB; Figure S1b‐ii, Supporting Information) and mass spectrometry (three biological replicas, MS; Figure S1b‐iii, Supporting Information). Lamin‐A and lamin‐B1 protein levels, as averaged across IF, WB and MS, are in agreement with RNA levels (three biological replicas of RNA‐Seq.; Figure S1c, Supporting Information). Nonzero RNA levels of knockout lamins originate from residual exon sequences that were upstream of cleavage site.^[^ 15, 38 ^]^ Consistently, nonzero protein levels may originate from nonspecific antibody interactions (IF and WB), lamin fragments and false positive readouts (MS). To study the mechanical contributions of chromatin in association with lamins, we treated WT, AKO, and TKO cells with Trichostatin‐A, which is a potent Class‐I and ‐II histone deacetylase inhibitor (HDACi). HDACi leads to a rapid and global increase in histone acetylation (Figure S2, Supporting Information), thus removing positive charges on lysine residues and relaxing the interaction with DNA (negatively charged phosphate groups).^[^ 43 ^]^ Collectively, we established a set of lamin‐knockout and rescue MEF cells that facilitate a thorough study of the contributions of lamin‐A and lamin‐B1 to nucleus mechanics and used HDACi to study the mechanical implications of chromatin condensation.

### 2.2 Measuring Nucleus Mechanics within Intact Cells over Biologically Relevant Length Scales

Physical stretching and squashing of the cell nucleus due to cell migration across narrow constrictions, mechanical load applied to skeletal tissues and similar physiologically relevant circumstances, typically deform a significant part of the nuclear volume and are not limited to micron‐to‐submicron regions of the nuclear envelope (NE).^[^ 1, 44 ^]^ To study such nucleus deformations in response to applied stresses, we employed micropipette aspiration (MPA), which is a simple, stable, and robust method for performing viscoelastic profiling of nuclei, cells, and tissues.^[^ 45, 46 ^]^

**Figure 1:**
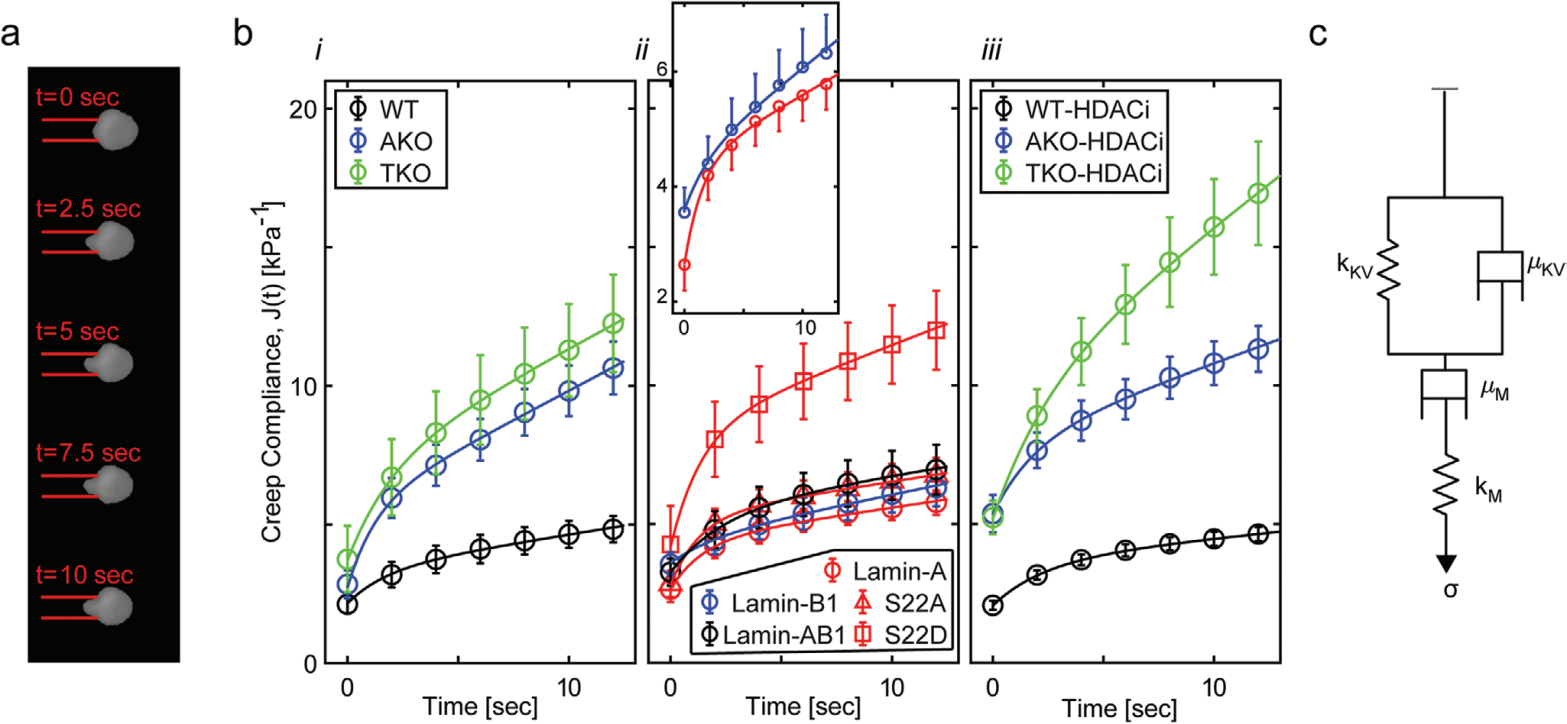
The cell nucleus responds to applied forces like a viscoelastic solid at short time and a viscoelastic fluid at long time: a) Representative time‐lapse images of nucleus aspiration into a pipette (6 µm inner diameter) at constant pressure. b) Creep compliance dynamics is plotted for i) wild‐type and lamin‐knockout cells, ii) lamin‐rescue in TKO cells, and iii) HDACi‐treated cells. In response to applied stress (turned on at *t* = 0), nuclei undergo an instantaneous elastic stretch followed by a viscoelastic deformation that approaches a steady state viscous flow. (ii‐inset) Zoom‐in of the creep compliance curves highlights the differences between A‐rescue and B1‐rescule nuclei. c) Creep compliance curves are fitted by the four‐element Burgers model (solid lines in b; R‐square fits >0.99). WT: wild‐type. AKO: Lmna knockout. TKO: Triple lamin genes knockout. S22D and S22A: Rescue of lamin‐A serine‐22 to aspartic acid and to alanine phosphomimetic site mutations, respectively. HDACi: histone deacetylase inhibitor drug. Lamin‐AB1: Rescue of lamin‐A and lamin‐B1 coexpression.

To prevent potential irreversible structural and mechanical alterations that may occur during nucleus isolation, MPA was applied to nuclei within intact cells. Cytoskeletal contributions were eliminated by treating the cells with actin depolymerizing drugs. A constant negative pressure was applied inside the pipette with cross sectional area that was approximately half the cross sectional area the nucleus (**Figure** **1**a). We applied ≈1 kPa step pressure and recorded nucleus aspiration dynamics for >12 s. Nucleus deformability was analyzed by calculating the creep compliance (*J*(*t*) = strain dynamics/stress) in accordance with the half‐space model and averaged across 10‐to‐20 cells per condition (Figure 1b).^[^ 47 ^]^ Stress intensity and duration are consistent with cell‐generated tractions^[^ 48, 49, 50 ^]^ and the discrete episodes of nuclear deformations (see Video S1, Supporting Information).^[^ 51 ^]^ However, the instantaneous application of stress by MPA and rate of induced heterogeneous deformations may deviate from physiological processes that deform the cell nucleus for example during constricted cell migration.^[^ 4, 6, 52 ^]^

### 2.3 A Viscoelastic Four‐Element Model of Nucleus Mechanics

Creep test measurements exhibit complex viscoelastic responses to applied stress that depend on lamin expression and phosphorylation, and chromatin decondensation (Figure 1b). However, nuclei across all conditions share the following characteristics as illustrated in Figure S3 (Supporting Information). Elastic response: The nucleus stretches instantaneously like a spring at *t* = 0 the moment stress is applied (*J*_0_). Viscoelastic stretching: The nucleus is aspirated viscoelastically into the pipette over a typical time scale τ. Viscous deformation: Nuclear creep approaches a constant rate α. This distinctive mechanical response was obtained both at low and high loading (Figure S4a, Supporting Information) and was also shared by mouse embryonic stem (mES) cells and induced pluripotent stem (iPS) cells, despite the marked differences in the organization of the nuclear lamina and chromatin compared with MEFs (Figure S5a, Supporting Information).^[^ 53, 54 ^]^

All relevant three‐element viscoelastic models failed to capture the main properties of nucleus response to applied forces across all conditions within the measured time range as described above (Table S2, Supporting Information). The minimal linear viscoelastic model that properly accounted for nuclear deformations across all conditions was the four‐element Burgers model (Figure 1c). In particular, the Maxwell and Kelvin representations of the SLS model are the only two three‐element models that capture instantaneous deformation (Table S2, Supporting Information, bottom). Hence, we tested their utility in modeling our MPA creep compliance curves and compared them to the Burgers model (Table S2, Supporting Information). The Burgers model provided the highest R‐square goodness of fit values not only because it consists of four elements. It also captured the steady state viscous deformation of the nuclei counter to the SLS models. Indeed, both SLS models showed poorer fits for nuclei that expressed lamin‐B but lacked lamin‐A owing to their long‐term low‐viscosity deformation as marked by high slope while retaining stiffness (Table S2, Supporting Information, marked in red).

Deformation dynamics of a Burgers material is described by creep compliance *J*(*t*) (strain/stress)

(1) $$J\;\left(t\right)=\frac{1}{k_{\text{M}}}+\frac{1}{k_{\text{KV}}}\left(1-e^{t/\tau }\right)+\frac{t}{\mu_{\text{M}}}$$

**Figure 2:**
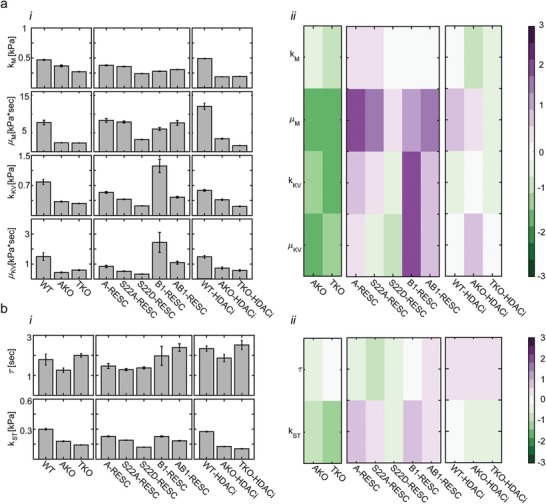
Viscoelastic characterization of the nucleus: a‐i) The viscosity and elasticity levels of the Burgers elements and ii) the log2‐fold ratios of lamin‐KO cells (relative to WT, left), lamin‐rescue cells (relative to TKO, middle), and HDACi‐treated cells (relative to nontreated cells, right) are presented for all MEF cells. b) Similarly, i) the viscoelastic response time τ and the steady‐state elasticity *k*_ST_ are reported, ii) as well as the log‐fold changes. *n* = 10 to 20 cells per condition. Error bars are the 95% fitting confidence bounds.

For each lamin and chromatin condition, we performed curve fitting of the experimental data (Figure 1b, Figures S4a and S5a, Supporting Information), evaluated the elasticity of the springs (*k*_M_, *k*_KV_) and the viscosity of the dashpots (μ_M_, μ_KV_), and calculated the physical parameters $\tau \;=\frac{\mu_{\text{KV}}}{k_{\text{KV}}}$ and $k_{\text{st}}=\frac{k_{\text{M}}\cdot k_{\text{KV}}}{k_{\text{M}}+k_{\text{KV}}}$ (**Figure** **2**a,b and Figures S4b, S5b, and S6b, Supporting Information). The R‐square goodness of fit scores of the averaged curves were greater than 0.99 (solid lines in Figure 1b) and ranged between 0.97 and 1 across the single nucleus creep compliance measurements (only a minority of measured nuclei showed 0.9–0.96 goodness of fit).

Short and long time regimes are defined by τ, which ranges between 1 and 2.5 s (Figure 2b‐ii and Figures S4b‐ii and S5b‐ii, Supporting Information). At short times (*t* < 1 s), nucleus creep compliance is approximated by an elastic response

(2) $$J_{t<\tau }\left(t\right)≅J_{0}=\frac{1}{k_{\text{M}}}$$

Here, nucleus stiffness is set by *k*_M_, which ranges between 0.27 kPa for TKO MEFs and pluripotent stem cells to 0.5 kPa for WT and lamin‐rescue MEFs (Figure 2a‐i and Figure S5b‐i, Supporting Information). At longer times scales (*t* > 2.5 s), the nucleus gradually creeps like a viscoelastic fluid

(3) $$J_{t>\tau }\left(t\right)≅J_{\infty }+\;\alpha t=\frac{1}{k_{\text{ST}}}+\frac{t}{\mu_{\text{M}}}$$

With time, the nucleus deforms continuously with a rate that is set by viscosity μ_M_ and resist applied forces with steady state stiffness set by *k*_st_. μ_M_ and *k*_st_ range between 2.3 kPa s and 0.15 kPa (TKO cells) and 9.5 kPa s and 0.25 kPa (WT and lamin‐rescue cells). A Burgers material response allows the nucleus to absorb applied impact via elastic stretching and dissipate continuous stresses by deforming viscoelastically to prevent tear and break of the genome and the lamina.^[^ 30 ^]^

Natural and synthetic biomaterials composed of cross‐linked filaments often exhibit nonlinear mechanics and specifically strain stiffening.^[^ 55 ^]^ To study nuclear behavior under different loading regimes, we performed MPA of WT and TKO cells using low (<0.8 kPa) and high (>1.6 kPa) aspiration pressure. A Burgers response was obtained at both regimes (Figure S4a, Supporting Information). Nuclear compliance decreased with increasing load. WT nuclei were fitted by higher stiffness terms *k*_M_, *k*_KV,_ and *k*_ST_ and viscous terms μ_M_, μ_KV_, yet the response time τ remained invariant (Figure S4b, Supporting Information). The WT versus TKO fold‐change ratios were conserved between low and high loading levels.

To further validate a Burgers response as observed by MPA, we employed nanoindentation rheology (Chiaro nanoindenter, Optics11). WT and TKO cells were allowed to adhere onto rigid glass surfaces and creep‐test was performed on the nuclei using *f* = 1 μN indentation force (see Video S2, Supporting Information). Creep compliance dynamics was calculated based on nucleus surface indentation measurements δ(*t*) (Figure S6a, Supporting Information). To account for the disk‐like geometry of the nucleus of adhering cells,^[^ 13 ^]^ we employed the Dimitiriadis approximation for non‐bonded thin substrates^[^ 56 ^]^

(4) $$\begin{array}{l} J\;\left(t\right)=\frac{16R^{1/2}\delta {\left(t\right)}^{3/2}}{9f}\;(1+1.133\chi \left(t\right)+1.283\chi {\left(t\right)}^{2}\\ \;\;\;\;\;\;\;\;\;\;\;\;\;\;\;\;\;\;\;\;\;\;\;\;\;\;\;\;\;\;\;\;\;\;\;\;\;\;\;\;\;\;\;\;\;+0.769\chi {\left(t\right)}^{3}+0.0975\chi {\left(t\right)}^{4}) \end{array}$$

Here we assumed negligible compressibility (ν = 0.5). Force *f* was applied via a spherical bead of radius *R*. $\chi (t)=\sqrt{R\delta (t)}\text{/}h$ is the dimensionless deformation coefficient set by nucleus thickness *h*  =  3 μm and radius *R* of the spherical bead probe. Despite the marked differences between nanoindentation and MPA rheology in terms of the size of deformations and loading geometry, a Burgers response was obtained also by nanoindentation creep test. Nanoindentation measurements involved up to ≈2 µm apical deformations, showing increased compliance of TKO nuclei (Figure S6a, Supporting Information). Consistently, the relationship between the Burgers parameters, τ and *k*_ST_ of WT and TKO cells as was obtained by MPA is conserved.

### 2.4 Stiffness is Controlled Both by Lamin‐A and Lamin‐B1—Viscosity Is Primarily Controlled by Lamin‐A

WT nuclei are relatively stiff and viscous. Knockout of lamin‐A/C (AKO and TKO cells) decreases μ_M_ approximately fourfold and rescue of lamin‐A in TKO cells (A‐rescue) increases μ_M_ also approximately fourfold (Figure 2a‐ii). This increase in viscosity is governed by S22A and not by S22D, namely it is an inherent property of the filamentous meshwork of A‐type lamins and not the nucleoplasmic pool of disassembled lamin‐A. In comparison with A‐rescue nuclei, the creep compliance curve of B1‐rescue nuclei appears to have a slightly higher instantaneous compliance and a steeper steady‐state slope (Figure 1b‐ii, inset). Indeed, the differences in instantaneous and steady state stiffness (*k*_M_ and *k*_ST_) are not statistically significant, yet the differences in nucleus viscosity (μ_M_) and response time (τ) show higher statistical significance (Table S1, Supporting Information), indicating that both lamins contribute to nucleus stiffness while viscosity is rendered mostly by lamin‐A. The expression of lamin‐B1 increases both *k*_KV_ and μ_KV_ fourfold. *k*_KV_ sets τ (τ  = μ_KV_/*k*_KV_ ) and contributes to steady‐state stiffness (in series with *k*_M_). Hence, fitting *k*_KV_ should satisfy both *k*_KV_ and τ. Since the instantaneous deformation of A‐rescue nuclei is lower yet steady‐state stiffness is comparable with B1‐rescue nuclei (see inset of Figure 1b‐ii), *k*_KV_ is set twofold higher in the latter case. μ_KV_ is set 2.5 fold higher to compensate for *k*_KV_ thus satisfying τ. The fact that setting of four parameters successfully fits the compliance curves validate the utility of the Burgers model of nucleus mechanics. Rescue of lamins barely affects *k*_M_ but steady‐state nucleus stiffness increases 1.6 fold either due to lamin‐A or lamin‐B1 expression (Figure 2b‐ii). The increase in μ_M_ in AB1‐rescue cells (coexpression of lamin‐A and lamin‐B1) is similar to A‐recue cells. In contrast, *k*_KV_ and μ_KV_ do not show the same increase in AB1‐rescue cells as in B1‐resuce cells despite their similar lamin rescue levels (Figure S1c, Supporting Information), suggesting that lamin‐A and lamin‐B1 are competing for binding the same chromatin sites. This demonstrates that the mechanical contributions of A‐ and B‐type lamins are nonadditive, consistent with the separation between the filamentous networks formed by A‐ and B‐type lamins and their binding competition of chromatin and inner nuclear membrane linkers.^[^ 8, 9, 57 ^]^

### 2.5 Linking Nucleus Mechanics and Gene Expression Profiles of Lamins

**Figure 3:**
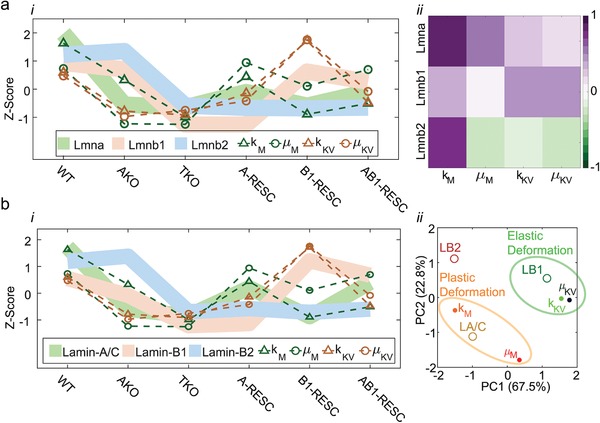
Expression profiles of Lamin‐A cluster with nucleus viscosity and Lamin‐B1 with nucleus stiffness. The standardized z‐score profiles of the four Burgers elements are plotted across all MEF nuclei together with the z‐score profiles of a) lamins' mRNA levels (RNA‐Seq) and b) lamins' protein levels (averaged across IF, WB and MS, Figure S1, Supporting Information. Changes in k_M and µ_M overlap with Lamin‐A/C (green) both at the a‐i) RNA and the b‐i) protein levels. Changes in k_KV and µ_KV weakly overlap with Lmnb‐1 (brown) at the mRNA and the protein levels. a‐ii) Pearson coefficients of correlation are calculated between lamin gene expression levels and the Burgers elements. Maximal correlations of k_M and µ_M are obtained with Lmna and of k_KV and µ_KV with Lmnb1. b‐ii) At the protein level, principle component analysis establishes associations between k_M and µ_M with Lamin‐A/C and between k_KV and µ_KV with Lamin‐B1. In accordance with the Maxwell module and the Kelvin‐Voigt module, lamin‐A/C dominates plastic deformation (orange) and lamin‐B1 dominates elastic deformation (green) of the nucleus in response to applied stress.

To evaluate the mechanical roles of lamin‐A and lamin‐B1, we study the relationship between lamin RNA and protein levels in WT, knockouts and rescue MEF cells and the corresponding viscoelastic Burgers elements. Lamin RNA levels were obtained via genome‐wide RNA‐Seq. (three biological replicas of each MEF type). The standard scores (z‐scores) of *Lmna* normalized across WT, TKO, A‐rescue, B1‐rescue and AB1‐rescue MEFs share the same trend as the z‐scores of the Maxwell elements *k*_M_ and μ_M_ whereas *k*_KV_ and μ_KV_ show partial overlap with *Lmnb*1 (**Figure** **3**a‐i). These molecular associations are quantified via Pearson correlation analysis (Figure 3a‐ii). *Lmna* positively correlates with *k*_M_ and μ_M_ (0.88 and 0.71, respectively) and *k*_KV_ and μ_KV_ positively correlate with *Lmnb*1 (0.27 and 0.23, respectively) and negatively with *Lmnb*2 (−0.39 and −0.53, respectively), yet *Lmnb*2 expression levels are very low in all cells. Compared with RNA levels, lamin protein levels exhibit even higher association with the Burgers elements (Figure 3b‐i). Here, we used the protein levels that were averaged across IF, WB and MS measurements, thus minimizing method‐specific bias (Figure S1c, Supporting Information). Lamin‐A z‐score protein profiles overlapped with *k*_M_ and μ_M_ (Pearson correlation scores 0.84 and 0.81, respectively) and lamin‐B1 overlapped with *k*_KV_ and μ_KV_ (Pearson correlation scores 0.59 and 0.62, respectively). Consistently, lamin‐A clustered with *k*_M_ and μ_M_ and lamin‐B1 clustered with *k*_KV_ and μ_KV_ in the principle component analysis of the normalized lamin profiles and the Burgers elements (Figure 3b‐ii). Consistent with the viscoelastic nature of the Maxwell and the Kelvin‐Voigt modules, our PCA clustering analysis associates lamin‐A with a plastic deformation closer than with elastic deformation and lamin‐B1 is associated with an elastic deformation more so than with plastic deformation

### 2.6 Linking Nucleus Mechanics and Chromatin

**Figure 4:**
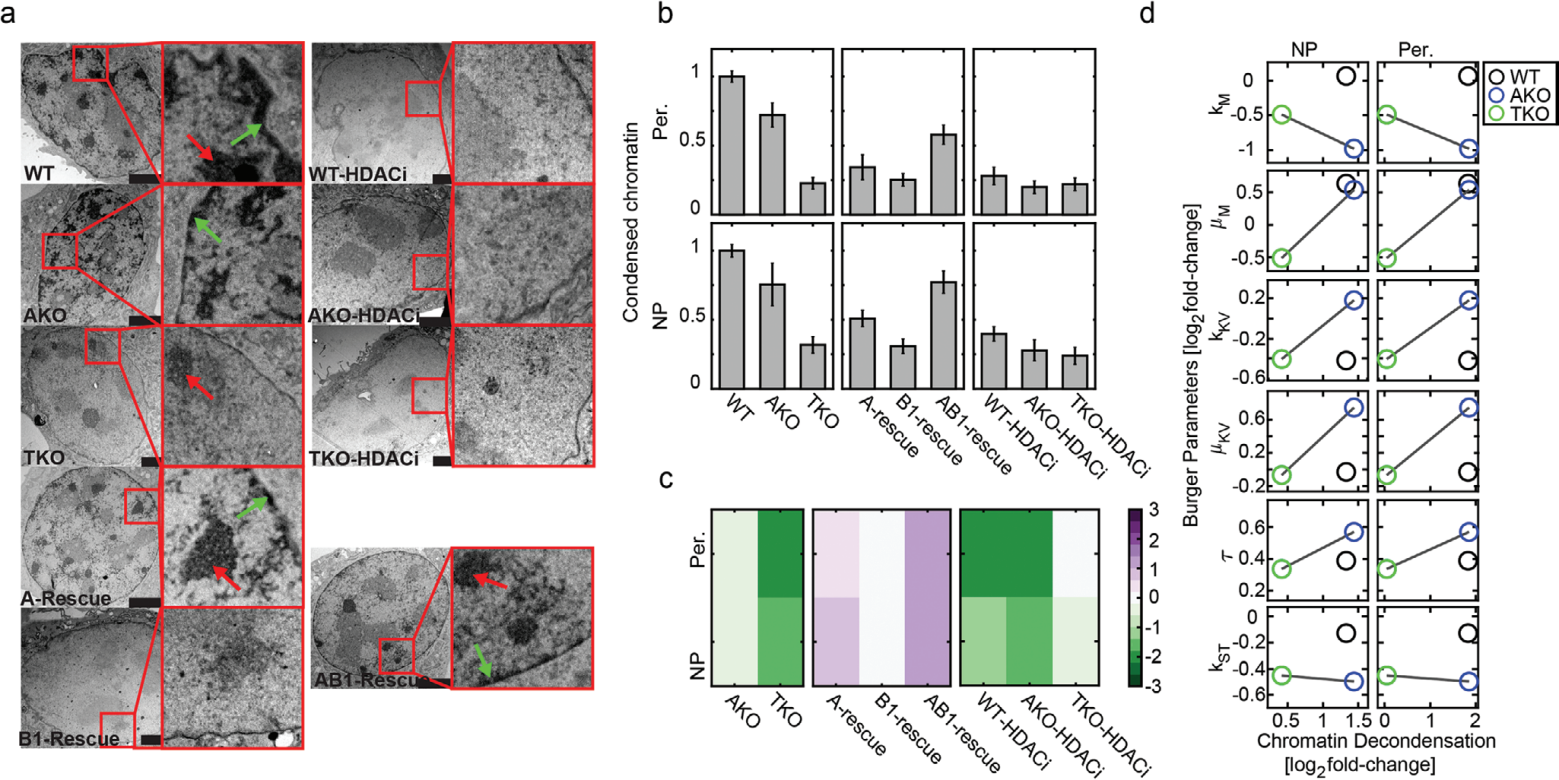
Condensed heterochromatin is stabilized by lamins and chromatin decondensation contributes to nucleus viscoeaslticity. a) Representative transmission electron micrographs of nuclei of WT, lamin‐knockout, lamin‐rescue, and HDACi‐treated cells highlight the differences in the intensity and width of the peripheral (Per., green arrows) and nucleoplasmic (NP, red arrows) condensed chromatin. TKO and HDACi‐treated nuclei show basal levels of condensed chromatin. b) Blinded scoring of condensed chromatin levels (three independent evaluators) and c) log2‐fold ratios of knockout cells (relative to WT), lamin rescue cells (relative to TKO) and HDACi‐treated cells (relative to nontreated). d) Log2 fold changes of the Burgers elements are plotted as a function of Log2 fold changes of nucleoplasmic (left) and peripheral (right) chromatin decondensation of WT (Lamin‐A expressing cells), AKO and TKO (Lamin‐A null cells) nuclei.

Condensed chromatin levels were evaluated via transmission electron microscopy (TEM, **Figure** **4**a and Figure S7, Supporting Information). Compacted chromatin, which is visualized as dark regions, is transformed into a more loosen structure following HDACi treatment. Indeed, there is a significant decrease in the intensity and width of peripheral condensed chromatin at the nuclear envelope (green arrows), accounting for the role of lamins in anchoring and stabilizing lamina‐associated domains (LAD's),^[^ 11, 16, 58, 59 ^]^ and a decrease in the number and size of condensed chromatin regions at the nucleoplasm in WT and AKO nuclei following HDACi treatment (Figure 4a). Centromeres appear as discrete foci of constitutive heterochromatin at the nucleoplasm (red arrows). Relative intensities of peripheral and nucleoplasmic condensed chromatin were scored unbiasedly in a blinded fashion independently by three examiners. Average scores were obtained across *n* = 3 to 16 nuclei per condition (Figure 4b). Log2 fold‐changes in chromatin condensation due to lamin knockouts (left), lamin‐rescue (middle) and HDACi treatment (right) are calculated (Figure 4c).

The mechanical properties of chromatin absent of lamin contributions are evaluated using TKO cells. TKO nuclei, in which chromatin is mostly decondensed (Figure 4ab), are soft ( *k*_M_ =  0.27 kPa; *k*_ST_ =  0.14 kPa) and deform with low viscosity (μ_M_ =  2.3 Pa s) relative to WT and AKO nuclei (Figure 2a‐i,b‐i). Consistent with their open chromatin configuration, HDACi treatment fails to further de‐condensate TKO chromatin, confirming that near‐basal heterochromatin levels are maintained in the absence of nuclear lamins (Figure 4c). Consistently, HDACi shows minor effect on TKO nucleus mechanics (Figure 2a‐ii,b‐ii). Compared with TKO nuclei, condensed chromatin levels are threefold higher in AKO nuclei and fourfold higher in WT nuclei. HDACi treatment of these cells decondenses chromatin to a near‐basal level (Figure 4c). To uncover the relationship between chromatin and nucleus mechanics we plot HDACi‐treated versus nontreated fold changes of the Burgers elements as a function of chromatin decondensation fold changes (Figure 4d). Most of the viscoelastic elements show small response to chromatin decondensation (Log2 fold change < |0.5|). However, the instantaneous and steady state stiffness elements *k*_M_ and *k*_ST_ soften with chromatin decondensation in TKO and AKO nuclei. However, chromatin decondensation of WT nuclei (lamin‐A expressing cells) fails to soften *k*_M_ and shows only a small decrease in *k*_ST_ (Figure 4d). Chromatin de‐condensation also increases viscosity (μ_M_) in WT and AKO nuclei and decreases viscosity in TKO nuclei that lack lamins.

We compare our rheological measurements with published MPA creep test results (**Table** **1**). In addition to creep compliance slope α and response time τ (Figure S3, Supporting Information), the effective instantaneous and steady state elastic moduli *E*_0_ and *E*_ST_ as extracted for each model are compared as well. The SLS model of a viscoelastic solid was applied to chemically and mechanically isolated articular chondrocyte nuclei.^[^ 31 ^]^ This SLS creep test asymptotically approaches finite deformation at very long time (*t* > 100 s). An effective description of the lamina using a Maxwell model consisted of an elastic spring (lamin‐B1) and a viscous dashpot (lamin‐A) connected in series, revealing a stoichiometric scaling relationship $\tau \sim {\left(\text{Lamins}\;\text{A}:\text{B}\right)}^{2.5}$.^[^ 14 ^]^ Despite the differences in cell type and nuclear isolation, our results are in agreement with previous measurements, thus confirming the generality of our model.

**Table 1:** Comparison of nuclear viscoelastic properties measured by creep test MPA

	SLS modela) ^[^ 31 ^]^	Maxwell^[^ 14 ^]^	Burgers model
α [(kPa s)^−1^]	0	0.12–0.5	0.1–0.5
τ [s]	20	0.3–30	1–3
*E*_0_ [kPa]	0.4–0.53	0.1–0.3	0.25–0.5
*E*_ST_ [kPa]	0.2–0.26	0.1–0.3	0.15–0.25

a) Elastic moduli were rescaled to account for differences in creep compliance geometric factor.

## 3 Discussion

The cell nucleus is as soft as a yogurt gel and the filamentous nuclear lamina is as viscous as a caulking compound (not to be confused with nucleoplasmic viscosity, which is significantly lower).^[^ 60 ^]^ In response to applied mechanical load, the cell nucleus deforms while providing the mechanical strength to protect the genetic material and maintain genome organization. Tumor cell transendothelial migration across sub‐nuclear spaces typically lasts several minutes.^[^ 61 ^]^ Here we probe nucleus resistance during 12 s of aspiration and identify an inherent response time range 1 < τ < 2.5. The fact that water content of the nucleus (≈80%)^[^ 62 ^]^ can squeeze out during constricted migration much faster (0.2 s)^[^ 63 ^]^ confirms that our measurements are performed at volumetric steady state (Figure 1 and Figures S4–S6, Supporting Information).

The mechanical properties of the nucleus vary between cell types and cellular states. To account for the differences that are associated with cell type, lamin expression and phosphorylation, and heterochromatin organization, a linear viscoelastic four‐element model was employed that captures viscous flow under load unlike a three‐element solid‐like model that was previously used.^[^ 31, 64 ^]^ This minimal model successfully captures the dynamic resistance to applied forces by all nuclei across all conditions (Figure 2) and cell types (Figure S5, Supporting Information) both at low and high loading (Figure S4, Supporting Information) and is reproduced by indentation‐based creep test rheology (Figure S6, Supporting Information). Our measurements account for deformations that extend over <25% of the nuclear volume and persist for <20 s. Hence, we do not exclude an elastic restraining element that resists larger deformations at later times as reported by Sato and co‐workers for endothelial cells^[^ 64 ^]^ and by Guilak and co‐workers for isolated nuclei.^[^ 31 ^]^ In this case, a three‐element viscoelastic model such as the SLS model would suffice. Moreover, this restraining element is consistent with strain stiffening of the A‐type lamin meshwork at large deformations as implied by our low and high loading measurements (Figure S4, Supporting Information) and as reported by Stephens et al.^[^ 29 ^]^

Our work highlights the complex coupling between lamins and chromatin in nucleus response to applied forces. Chromatin fills in the entire nucleoplasmic volume and interacts with nucleoplasmic crosslinkers^[^ 65 ^]^ and with nuclear envelope proteins.^[^ 66 ^]^ Hence, heterochromatin decompaction is expected to soften the nucleus. The effects of chromatin decondensation on nucleus mechanics have been studied in various cell types using multiple microrheological approaches. Chalut et al. reported on nucleus softening upon chromatin decondensation using HDACi in mES cells that lack lamin‐A/C,^[^ 67 ^]^ which is consistent with softening of AKO nuclei (Figure 2). Other studies induced small deformations using AFM and needle‐based micromanipulation to demonstrate nucleus softening upon chromatin decondensation in lamin‐A expressing cell lines.^[^ 26, 28, 68 ^]^ Stephens et al. also reported on softening of the nucleus due to chromatin decondensation but only for small deformations.^[^ 29 ^]^ However, no significant softening is reported for large deformations > 3 µm—in agreement with our MPA measurements of HDACi‐treated WT cells (Figure 2). Other studies indirectly manipulated chromatin by increasing the concentrations of divalent ions^[^ 12, 32 ^]^ or by introducing DNA intercalating agents and digesting nucleases.^[^ 29 ^]^ Here we perturbed cellular mechanisms by targeting endogenous histone deacetylases that regulate heterochromatin compaction.^[^ 43 ^]^ The fact that WT nuclei do not soften in response to chromatin decondensation but not AKO or TKO nuclei (Figure 4), at least with respect to large deformations induced by MPA, arguably suggests that lamin‐A directly or indirectly stabilizes interactions with newly formed open chromatin segments both in the nucleoplasm and at the nuclear envelope.^[^ 69, 70, 71 ^]^ Acetylated euchromatic nanoclusters have been shown to interact with lamin‐A at the nucleoplasm^[^ 72 ^]^ in association with Lap2α,^[^ 65, 73 ^]^ thus contribute to maintaining nucleus stiffness. Uncovering lamin‐A interactions with decondensed chromatin is a fundamental property of nucleus organization and gene regulation, however it stems beyond the scope of this work.

The response time τ is the most robust viscoelastic parameter that changes the least across all lamin and chromatin conditions and across cell type (coefficient of variation CV  =  25%). τ is 1.8 s for WT MEFs and 2 s for mES and iPS cells, which lack lamin‐A and whose chromatin is to a great extent in open configuration similar to TKO MEFs (Figure 2b and Figure S5b, Supporting Information). At *t* = 0 s, the cell nucleus stretches elastically and this instantaneous stiffness (*k*_M_) changes little relative to other viscoelastic elements. The most significant perturbations are rescue of lamin‐A (specifically the nonphosphorylated S22A derivative) that stiffens *k*_M_ and chromatin de‐condensation in lamin‐A null nuclei that softens *k*_M_ (Figure 2a). Lamin‐A anchors and stabilizes heterochromatin at the nuclear envelope^[^ 11 ^]^ and forms weak and transient interactions with nucleoplasmic chromatin.^[^ 70, 71 ^]^ Indeed, we confirm that rescue of lamin‐A increases condensation of both peripheral and nucleoplasmic chromatin (Figure 4a–c). Expression of lamin‐B1 resists nucleus deformation between 0 < *t* < 3 s through the Kelvin‐Voigt elements that render solid‐like behavior. Unlike A‐type lamins, lamin‐B1 is localized to the nuclear envelope.^[^ 74 ^]^ Hence, stiffening of *k*_KV_ can be associated with chromatin interactions only at the nuclear envelope. Bases on our TEM analysis, lamin‐B1—chromatin interactions appear not to significantly promote chromatin compaction (Figure 4a–c). Indeed, chromatin decondensation of WT, AKO, and TKO nuclei has a small effect on *k*_KV_ that is not correlated with the degree of decondensation (Figure 4d). Nucleus resistance to a continuously applied force (*k*_ST_; *t* > 3) is equally rendered by lamin‐A and lamin‐B1 (Figure 2). Knockout of A and B lamins soften *k*_ST_ and rescue of A and B lamins stiffen *k*_ST_, yet lamin‐A and lamin‐B1 contribute to steady state stiffness through modifying *k*_M_ and *k*_KV_, respectively. Consistent with *k*_M_, chromatin decondesation softens AKO and TKO nuclei but not WT nuclei. In addition to elastic response to applied forces, the nucleus also flows with viscosity μ_M_. Lamin‐A is the dominant component that restrains flow rate. Viscosity is also altered by chromatin decondensation, yet this effect is relatively small (Figure 2a). Hence, low lamin‐A will decrease nucleus stiffness and will also permit rapid deformation, for example in granulocytes and metastatic cancer cells that transmigrate across narrow and rigid connective tissue boundaries.^[^ 75, 76, 77 ^]^ High lamin‐A will restrain deformation rate and strengthens the nuclear envelope and the nuclear interior (Figure 2), thus rendering mechanical strength to musculoskeletal cell nuclei that are constantly under load.^[^ 14 ^]^

**Figure 5:**
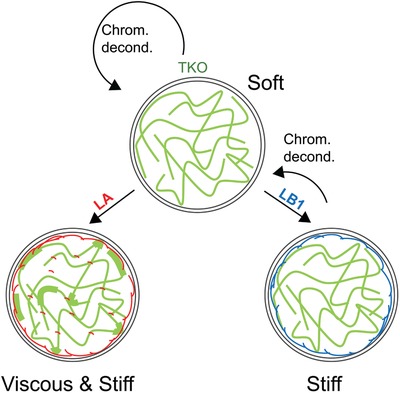
A simplified viscoelastic model of the nucleus: This simplified model captures only the dominant viscoelastic contributions of lamins and chromatin. Top: TKO nuclei (top) that lack all lamins and consist mainly of decondensed chromatin are soft (*k*_ST_ =  150 Pa) similar to mES and iPS nuclei and flow with relatively low viscosity ( μ_M_ =  2300 Pa s). Expression of lamin‐A or lamin‐B1 stiffen the nucleus whereas chromatin decondensation softens the nucleus in cells that lack lamin‐A (right). Lamin‐B1 contributes only to steady state stiffness *k*_ST_ and not to instantaneous stiffness *k*_M_. Left: Nucleus viscosity μ_M_ is dominated by lamin‐A (rescue of 30% lamin‐A WT levels leads to ≈3.5‐fold increase in μ_M_). Chromatin decondensation also increases viscosity of lamin‐A expressing nuclei. Coexpression of lamin‐A and lamin‐B1 increases both nucleus elasticity and viscosity and stabilizes chromatin condensation.

With the overall goal of providing a concise description of nucleus resistance to applied forces, we propose a simplified model that accounts only for the dominant viscoelastic contributions of A‐type lamins, B‐type lamins, and chromatin (**Figure** **5**). Our work thus provides a quantitative and unified framework for assessing nuclear deformation dynamics and for predicting the subsequent phenomenology based on changes in lamin expression and chromatin organization that are associated with embryonic developmental, tissue regeneration, malignant transformation, and disease (laminopathies).

## 4 Experimental Section

##### MEF Cells and Cell Culture

Triple knockout (TKO) mouse embryonic stem cells (mES) cells that are *Lmna, Lmnb1 and Lmnb2* nulls were generously contributed by the Zheng lab (Carnegie Institution, Washington DC, USA).^[^ 15, 38, 78 ^]^ TKO mES cells were injected into mouse blastocysts and transferred into recipient female mice (Hebrew University Institutional Ethics Committee – Research Number MD‐14‐14057‐4 and MD‐15‐14360‐4). Mice were sacrificed on day E13.5, TKO mouse embryonic fibroblasts (MEF's) were harvested and screen based on antibiotic resistance motif (G418, 300 µg mL^−1^), and a spontaneously immortalized TKO MEF cell line was generated via repeated passaging in culture. Immortalized wild‐type (WT) MEF cell line was generated similarly. Immortalized *Lmna* knockout (AKO) MEF cell line that was generated by the Misteli lab (Center for Cancer Research, National Cancer Institute, NIH, Bethesda MD) was also included in this study.^[^ 42 ^]^

##### Cloning of Lamin‐Rescue Constructs

Mouse *Lmna* and *Lmnb1* cDNA sequences were cloned into a modified pInducer20 lentiviral vector (Addgene). Lamins were expressed under the doxycycline inducible (2 µg mL^−1^) Tet‐on promoter and c‐terminus fused to mCherry (Lamin‐A) and Venus (Lamin‐B1) fluorescent reporter proteins via the Thosea asigna virus 2A (T2A) self‐cleaving peptide. Phosphomimetic constructs were generated via site‐directed mutagenesis of lamin‐A Serine‐22 (Ser22) residue. Serine was exchanged with either aspartic acid (S22D) or alanine (S22A) to generate permanently phosphorylated or nonphosphorylatable constructs, respectively.

##### Generation and Culture of Lamin‐Rescue MEF Cells

Stable lamin‐rescue MEF cell lines were generated by infecting TKO cells with lentiviral lamin‐A‐T2A‐Venus and the associated Ser22 phosphomimetic constructs, lamin‐B1‐T2A‐mCherry, or cotransfected with both lamin constructs. Lentiviral transfection protocol was performed as follows. Virus essentials solution was prepared by mixing 750 µL Optimem (31985‐047,Gibco), 12.5 µg of the lamin constructs, 4.5 µg PHR, 0.5 µg VSVG, 0.5 µg REV, and 0.5 µg Tat, 60 µg of PEI. Mixture was vortexed and left for 30 min at room temperature. Human embryonic kidney (HEK) 293T cells were cultured in 10 mL MEF medium supplemented with the virus essentials solution and incubated for 24 h. Medium was then exchanged and the supernatant was filtered on the next day (61004103,CA:0.45,SartoriusStedim), supplemented with polybrene (1:1000 v/v dilution) and added to a culture of MEF's. Lentiviral infection was repeated on the following day and medium was exchanged after 24 h. Transfected cells were FACS sorted. Nucleus staining of WT, AKO, TKO and rescue cell lines was performed via lentiviral transfection with H2B‐Orange. MEF's were cultured in Low Glucose DMEM (01‐050‐ 1A,Biological Industries) supplemented with 1% penicillin–streptomycin (03‐031‐1B,Biological Industries), 1% lalanyl‐l‐glutamine (03‐022‐1B,Biological Industries), 1% nonessential amino acids (NEAA, 01‐340‐1B, Biological Industries) and 15% fetal bovine serum (04‐127‐1,Biological Industries). MEF's were passaged once 80% confluence was reached. Infection rate was verified by fluorescence imaging following Dox addition (2 µg mL^−1^).

##### Culture of mES and iPS Cells

R26 mES cell line was derived from a cross between C57BL/6Rosa26‐M2rtTA and C57BL/6:Rosa26‐tdTomatoL‐S‐L. R26 mES cell line and iPS clones were cultured in DMEM supplemented with 10% FBS, 1% nonessential amino acids, 2 × 10^−3^ m l‐glutamine, in‐house mouse Leukemia inhibitory factor (mLif), 0.1 × 10^−3^ m b‐mercaptoethanol (Sigma), and antibiotics with 2i‐ PD0325901 (1 × 10^−3^ m) and CHIR99021 (3 × 10^−3^ m) (PeproTech).

##### HDACi Treatment and Staining of H4 Acetylation

HDACi treatment was performed by treated the cells with 100 ng mL^−1^ TSA (Sigma T8852) for 24 h. For immunofluorescence staining, cells were fixed with 4% paraformaldehyde for 10 min and permeabilized for 5 min in 0.5% Triton X‐100/PBS. Slides were rinse and incubated in PBS with 0.1% Tween 20, blocked with 10% fetal bovine serum and incubated with anti‐acetyl histone H4 antibody (Upstate, 06866) for 1 h at room temperature. After three consecutive 5 min washes in 0.1% Tween 20/PBS, cells were incubated for 1 h with secondary antibody (Rrx donkey‐anti‐rabbit) and DAPI followed by three additional washes before mounting. Images were recorded on a Nikon TE‐2000 inverted microscope. Identical camera and microscope settings were maintained to facilitate intensity quantification of control and treated cells using a custom MATLAB script. Intensities were normalized by DAPI intensity.

##### Generation of iPS Cells

Replication‐incompetent lentiviruses containing reprogramming factors (OSKM 3:3:3:1) were packaged with a lentiviral packaging mix (7.5 µg psPAX2 and 2.5 µg pGDM.2) in 293T cells and collected 48, 60, and 72 h after transfection. The supernatants were filtered through a 0.45 µm filter, supplemented with 8 mg mL^−1^ of polybrene (Sigma), and then used to infect MEFs. Six hours following the third infection, medium was replaced with fresh DMEM containing 10%FBS. Eighteen hours later, medium was replaced ESC reprogramming medium (DMEM supplemented with 10%FBS, 0.1 × 10^−3^ m b‐mercaptoethanol, 2 × 10^−3^ m l‐glutamine, 1%nonessential amino acids, in‐house mouse Leukemia inhibitory factor (mLif), and 2 mg mL^−1^ doxycycline). iPSC reprogramming medium was replaced every other day for 14 d, followed by 5 d in 2i/L culturing medium without dox. Separate stable colonies were isolated and propagated for further analysis.

##### Micropipette Aspiration

Nucleus mechanics was measured using a manometer‐based micropipette aspiration system made in house using a manual hydraulic micromanipulator (Nikon Narishige) and a pressure transducer (Validyne, Northridge CA, USA).^[^ 79 ^]^ Pipettes were pulled and forged to 3–6 µm inner diameter tips (Sutter Instruments MicroPuller P‐1000 and Narishige Micro‐Forger MF‐830). Pipettes were immersed in BSA to prevent cell adhesion to inner wall. To eliminate the mechanical contributions of the surrounding cytoskeleton, cells were trypsin‐detached, resuspended in MEF medium supplemented with Cytochalasin‐D (1 µg mL^−1^) and incubated for 30 min inside a humidified cell culture incubator at 37 °C, 5% CO_2_. Suspended cells were then placed on a glass‐bottom plate and mounted onto an inverted fluorescent microscope (Nikon Eclipse, Ti‐U). The pipette tip was brought into contact with the cell nucleus and basal suction pressure was applied to stably engage it. Using the H2B‐Orange fluorescent channel, nucleus aspiration dynamics was recorded (Andor Zyla sCMOS controlled via Nis Elements 4.50.00 command software) in response to stably applied ≈1 kPa suction inside the pipette relative to basal level.

The mechanical properties of the nuclei were evaluated based on the relationship between aspiration length *L*_p_(*t*) and applied suction *∆P* using the half‐space model. The creep compliance becomes^[^ 45, 47 ^]^

(5) $$J\;\left(t\right)\;=\;\frac{2\pi \phi L}{3R_{\text{p}}\Delta P}$$

where *R*_p_ is the inner pipette radius and ϕ ≈ 2 is the geometrical wall factor. In line with Sato et al.,^[^ 64 ^]^ the viscoelastic elements of the Burgers model are obtained by fitting *J*(*t*) using Equation 1.

##### Nanoindentation

Creep compliance test was performed by nanoindentation (Chiaro, Optics11) force using spherical probe of radius 41 µm and stiffness 45 [N m^‐1^]. Cells were allowed to adhere for 24 h onto glass surfaces and indentation was positioned above the fluorescently labeled nucleus as described above. Load‐controlled indentation allowing 1 s force ramp reaching at 1 µN force that was maintained for 10 s. Indentation and loading dynamics were probed and automatically calculated.

##### Immunofluorescence and Immunoblotting

Immunofluorescence was performed on 18 mm glass coverslips. Coverslips were immersed overnight at 37 °C in type1 collagen (0.2 mg mL^−1^ collagen Type1 (CORNING, 354236) and equal volume of 0.1 acetic acid (GADOT, 830168275) mixed in 50 × 10^−3^ m HEPES pH 8 (SIGMA, H3375‐100G)). Coverslips were rinsed in PBS and sterilized in UV for 3 h. For each condition, 10 000 cells were seeded on collagen coated coverslips in six‐well plate wells and rinsed in PBS after 4 h to remove nonadherent cells. After 24 h incubation, cells were fixed (15 min in 3.5% formaldehyde), rinsed with PBS, permeabilized (10 min in 0.5% Triton‐X), rinsed again in PBS and blocked for one hour (2% bovine serum albumin, BSA, SIGMA A7906). Immunostaining was performed in 2% BSA according to manufacturer's instructions.

For Western‐blotting, cells of all conditions were expanded on 10 cm culture plates. Once 85% confluence was reached, cells were harvested using trypsin (03‐050‐1A, Biological industries) and 1/8 was removed for calibration of protein content using a Bradford assay (B6916, Sigma). Harvested cells were centrifuged at 300G for 5 min to remove supernatant. Cell pellets were immersed in 1× lysis buffer at 90 °C (50 × 10^−3^ m tris pH 6.8 (Biolab Chemicals), 2.5% β‐mercaptoethanol (M6250, SIGMA), 2% sodium dodecyl sulfate (62862, Riedel‐de‐Haen), 0.01% bromophenol blue (B5525, SIGMA), and 10% glycerol (Romichol, 19‐557401‐33), in DDW), mixed and incubated for 3 min. DNA was fragmented via sonication and reheated to 90 °C for 3 min. Lysates were then vortexed, centrifuged for 10 min and stored at −80 °C. 20 µg total protein of each sample was loaded onto a 4–20% Gebagel (15G‐0420‐10, Geba LTD). Electrophoresis (IL128, Geba, LTD) was performed in 1× running buffer (25 × 10^−3^ m tris (002009239100, Bio‐Rad), 192 × 10^−3^ m Glycine (808822, MD) and 0.1% sodium dodecyl sulfate (62862, Riedel‐de‐Haen) in DDW) at 170‐200 V to reach size‐based separation. Gel was transferred onto a PVDF membrane (1704156, Bio‐Rad) using a dedicated blotting device (690BR011510, Bio‐Rad). Membranes were rinsed in 1× TBS/T (19 × 10^−3^ m tris (20092391, Biolab Chemicals), 137 × 10^−3^ m NaCl (19030591, Biolab Technologies) and 1:10,000 Tween 20 (X251‐07, J.T.Baker) in DDW) and blocked in a blocking buffer (1× TBS/T, 10% skim milk (232100, BD)) for 60 min. Immunoblotting was performed by immersing the membranes with primary antibodies diluted in 1% skim milk in TBS/T for 1 h (at room temp) or overnight (at 4 °C) according to manufacturer instructions. Membranes were rinsed in 1× TBS/T and immersed with secondary antibodies in 1% skim milk in TBS/T for 60 min. Band chemiluminescence signals were imaged (FUSION FX, VILBER), measured using ImageJ and normalized to background intensity.

Primary antibodies used for immunofluorescence and immunoblotting are anti lamin‐A (4C11, Cell Signaling, IF and WB), anti lamin‐B1 (Ab16048, Abcam, IF) and (Ab133741, Abcam, WB), and anti lamin‐B2 (Ab151735, Abcam, IF). Secondary antibodies are donkey anti‐mouse (Ab150109, Abcam, IF), donkey anti‐rabbit (Ab150067, Abcam, IF), goat anti‐mouse (115‐035‐003, Jackson ImmunoResearch, WB) and goat anti‐rabbit (111‐035‐003, Jackson ImmunoResearch, WB).

##### RNA Sequencing

RNA extraction was performed following Qiagen RNeasy Mini Kit (cat No's 74104 and 74106). Briefly, cells of each condition were harvested from one 10 cm plate each using trypsin, centrifuged to remove supernatant and lysed in RLT buffer containing 1% beta‐mercaptoethanol in accordance with plate confluency. RNA was extracted using RNeasy spin column according to manufacturer's instructions. DNA was degraded using RQ1 RNase‐Free DNase kit (Promega, M610A) according to manufacturer's protocol. Samples were eluted in RNase free water and stored at −80 °C.

Biological triplicates of extracted RNA were submitted to the Crown Institute for Genomics, Weizmann Institute of Science Israel. Libraries were prepared using the Genomics in house protocol for mRNA‐seq. Briefly, the polyA fraction (mRNA) was purified from 500 ng of total RNA followed by fragmentation and synthesis of double‐stranded cDNA. Then, end repair, A base addition, adapter ligation and PCR amplification steps were performed. Libraries were evaluated by Qubit (Thermo Fisher Scientific) and TapeStation (Agilent). Sequencing libraries were constructed with barcodes to allow multiplexing of 24 samples in two lanes. About 22 million single‐end 60‐bp reads were sequenced per sample on Illumina HiSeq 2500 V4 instrument. Transcript reads were validated using FastQC and aligned using BOWTIE against hg19 indices. Gene abundance profiles were evaluated based on assembled transcripts using Cufflinks. Gene expression profiles, depleted of ribosomal RNA, were normalized to mean gene count across all cell lines. Technical variability was removed by fitting the log squared correlation of variations (CV2) relative to the log mean expression of all genes across each cell line was fitted to a squared polynomial function and outlier genes were removed.

##### Proteomic Analysis (Mass Spectrometry)—Nucleus Isolation

MEF's were harvested using Trypsin (Biological Industries, 03‐050‐1A) and centrifuged at 300 g for 5 min to remove excess medium. Samples were rinsed in ice cold PBS (Biological Industries, 02‐023‐1A), centrifuged in ice cold Buffer 1 (Digitonin buffer: 150 × 10^−3^ m NaCl, 50 × 10^−3^ m HEPES pH 7.4, 25 µg mL^−1^ digitonin (#D141, SIGMA), 1% Protease inhibitor (#P9599, SIGMA)), and left on ice for 10 min. The supernatant that includes the cytosolic fraction was removed. The mixtures were centrifuged at 2000 g at 4oc for 10 min. Pellets were rinsed in ice cold PBS to remove left over Digitonin. Pellets were resuspended in 500 µL of ice cold Buffer 2 (NP40 Buffer: 150 × 10^−3^ m NaCl, 50 × 10^−3^ m HEPES pH 7.4, 1% NP40 (21‐3277, SIGMA)), incubated for 30 min and centrifuged at 7000 g at 4oc for 10 min. Supernatant that includes the membrane fraction of the cells was removed. Left over pellet was rinsed in 1 mL of ice‐cold PBS and centrifuged twice to remove excess NP40. Pellet was resuspended in 500 µL of ice cold buffer 3 (RIPA Buffer, 150 × 10^−3^ m NaCl, 50 × 10^−3^ m HEPES pH 7.4, 0.5% sodium deoxycholate, 0.1% sodium dodecyl sulfate (SDS), Benzonase (SIGMA #E1014, 1 µL mL^−1^, added just before use)) and placed on ice for 1 h to facilitate complete solubilization of nuclear proteins and digestion of genomic DNA. The samples were centrifuged at 7000 g at 4oc for 10 min and supernatant consisting of the nuclear fraction of proteins was removed and submitted for mass spectrometry proteomic profiling. Purity of the nuclear fractions was validated by immunoblotting showing lack of calreticulin and presence of H2B markers of membrane and nuclear fractions, respectively.

##### Proteomic Analysis (Mass Spectrometry)—Sample Preparation

Proteomic analysis was performed by The De Botton Protein Profiling institute of the Nancy and Stephen Grand Israel National Center for Personalized Medicine, Weizmann Institute of Science. All chemicals were from purchased Sigma Aldrich, St. Louis MO, USA, unless stated otherwise. Nuclear fractions volume was reduced to 200 μL using a 3 kDa MWCO filter (Millipore, Darmstadt, Germany) and 50 µg of total protein were removed for tryptic digest. Samples volume was adjusted to 20 μL comprising 50 × 10^−3^ m ammonium bicarbonate, digested with trypsin using S‐strap (Protifi, Huntington NY, USA) according to the manufacturer's instructions, vacuum dried and stored in −80 °C until analysis.^[^ 80 ^]^

##### Proteomic Analysis (Mass Spectrometry)—Liquid Chromatography

ULC/MS grade solvents were used for all chromatographic steps. Each sample was loaded using split‐less nano‐ultra performance liquid chromatography (10 kpsi MClass; Waters, Milford, MA, USA). The mobile phase was: a) H_2_O + 0.1% formic acid and b) acetonitrile + 0.1% formic acid. Desalting of the samples was performed online using a reversed‐phase Symmetry C18 trapping column (180 µm internal diameter, 20 mm length, 5 µm particle size; Waters). The peptides were then separated using a T3 HSS nanocolumn (75 µm internal diameter, 250 mm length, 1.8 µm particle size; Waters) at 0.35 µL min^−1^. Peptides were eluted from the column into the mass spectrometer using the following gradient: 4% to 30%B in 155 min, 30% to 90%B in 5 min, maintained at 90% for 5 min and then back to initial conditions.

##### Proteomic Analysis (Mass Spectrometry)—Mass Spectrometry

The nanoUPLC was coupled online through a nanoESI emitter (10 µm tip; New Objective; Woburn, MA, USA) to a quadrupole orbitrap mass spectrometer (Q Exactive Plus, Thermo Scientific) using a FlexIon nanospray apparatus (Proxeon). Data were acquired in data dependent acquisition (DDA) mode, using a Top10 method. MS1 resolution was set to 70 000 (at 400 m/z), mass range of 300–1650 m/z, AGC of 3e6 and maximum injection time was set to 60 ms. MS2 resolution was set to 17 500, quadrupole isolation 1.7 m/z, AGC of 1e6, dynamic exclusion of 60 s and maximum injection time of 60 ms.

##### Proteomic Analysis (Mass Spectrometry)—Data Processing and Analysis

Raw data were processed in Maxquant version 1.6.0.16. Data were searched against the SwissProt murine database (March 2017 version) appended with common laboratory contaminant proteins. Fixed modification was set to carbamidomethylation of cysteine and variable modifications were set to protein N‐term acetylation, oxidation of methionine and deamidation of asparagine and glutamine. Search results were filtered to achieve maximum false discovery rate of 1% at the protein level. Protein LFQ intensities were calculated based on razor and unique peptides. The LFQ and iBAQ values were further processed in Perseus version 1.6.0.7. A Student's t‐test, after logarithmic transformation, was used to identify significant differences in LFQ intensities across the biological replica. Fold changes were calculated based on the ratio of means of the different samples.

## Conflict of Interest

The authors declare no conflict of interest.

## Supporting information

Supporting Information
