# Supplementary material for: A Unified Linear Viscoelastic Model of the Cell Nucleus Defines the Mechanical Contributions of Lamins and Chromatin
Source: Adv Sci (Weinh). 2020 Mar 5;7(8):1901222. doi: 10.1002/advs.201901222 (PMC7175345; doi:10.1002/advs.201901222)
Supplement: Supplementary file 1 — Supporting Information [file ADVS-7-1901222-s001.pdf]

Copyright WILEY-VCH Verlag GmbH & Co. KGaA, 69469 Weinheim, Germany, 2020.

ADVANCED SCIENCE
Open Access

Supporting Information

for *Adv. Sci.,* DOI: 10.1002/advs.201901222

A Unified Linear Viscoelastic Model of the Cell Nucleus Defines the Mechanical Contributions of Lamins and Chromatin

*Oren Wintner, Nivi Hirsch-Attas, Miriam Schlossberg, Fani Brofman, Roy Friedman, Meital Kupervaser, Danny Kitsberg, and Amnon Buxboim**

Supplementary figures

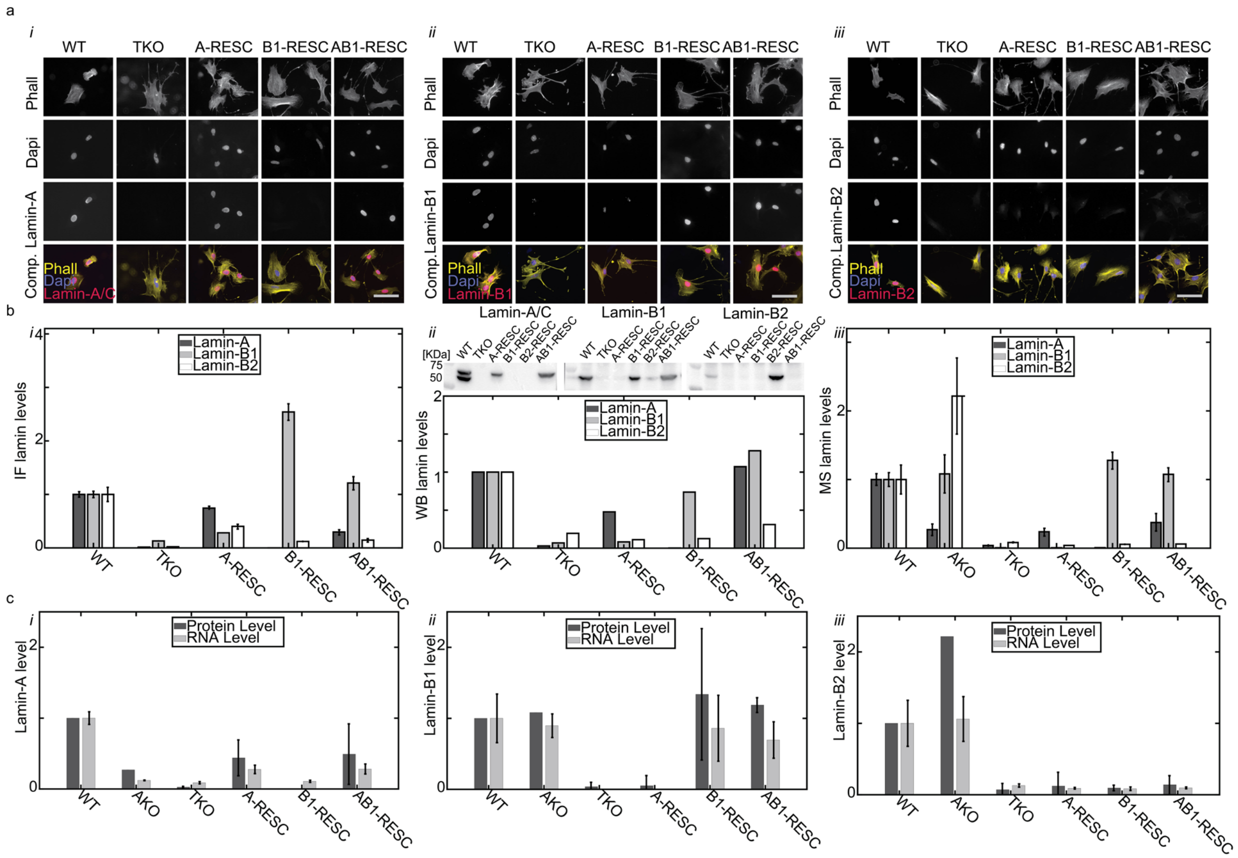

Figure S1 | Rescue of lamins with controlled expression levels: (a) Representative immunofluorescence images of MEF cells immunostained against (i) lamin-A, (ii) lamin-B1, and (iii) lamin-B2. (b) Relative lamin-A, lamin-B1 and lamin-B2 expression levels quantified by (i) immunofluorescence **(n>30 per condition)**, (ii) immunoblotting, and (iii) mass spectrometry. Residual lamin levels reported by immunofluorescence and immunoblotting for non-expressed genes are due to nonspecific interactions of the antibodies. (c) RNA levels (RNA-Seq.) and protein levels of (i) lamin-A/C, (ii) lamin-B1 and (iii) lamin-B2 are reported for WT, knockout and rescue MEF cells. Protein levels of lamins rescue ranges between 50% to 130% of wildtype levels. RNA levels are averaged across three biological replica and protein levels are averaged across immunofluorescence, immunoblotting and mass spectrometry measurements. Error-bars: standard error of the mean.

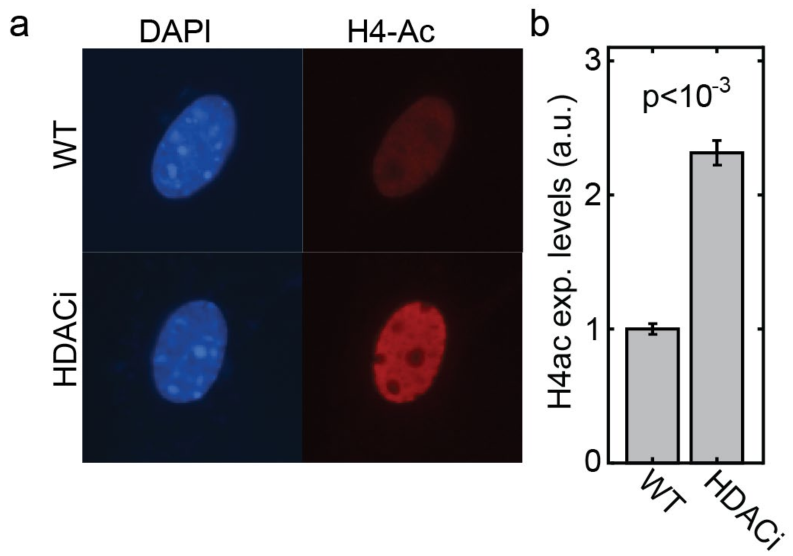

Figure S2: HDACi increases histone acetylation. (a) Immunofluorescence of H4 acetylation is shown for a WT MEF cell and a cell treated with HDACi. (b) HDACi increases acetylation 2.5 fold.

6

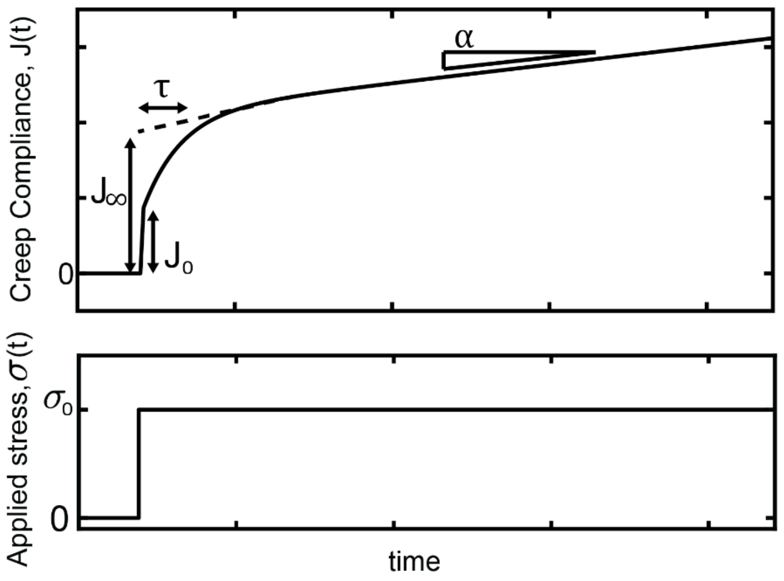

Figure S3 | Viscoelastic properties of the four-elements Burgers material: The creep compliance, $J(t)$, of a Burgers material (top) is illustrated in response to an applied stress step $\sigma(t)$ (bottom). The Maxwell spring stretches instantaneously, $J_0 = \frac{1}{k_M}$, followed by a damped viscoelastic deformation of the Kelvin-Voigt module over a time scale $\tau = \frac{\mu_{KV}}{k_{KV}}$. At long times, $t > \tau$, the effective steady state elastic resistance is set by the Maxwell and Kelvin-Voigt springs connected in series: $k_{ST} = \frac{k_M \cdot k_{KV}}{k_M + k_{KV}}$. The Burgers material continues to deform in a purely viscous manner as characterized by slope $\alpha = {\mu_M}^{-1}$.

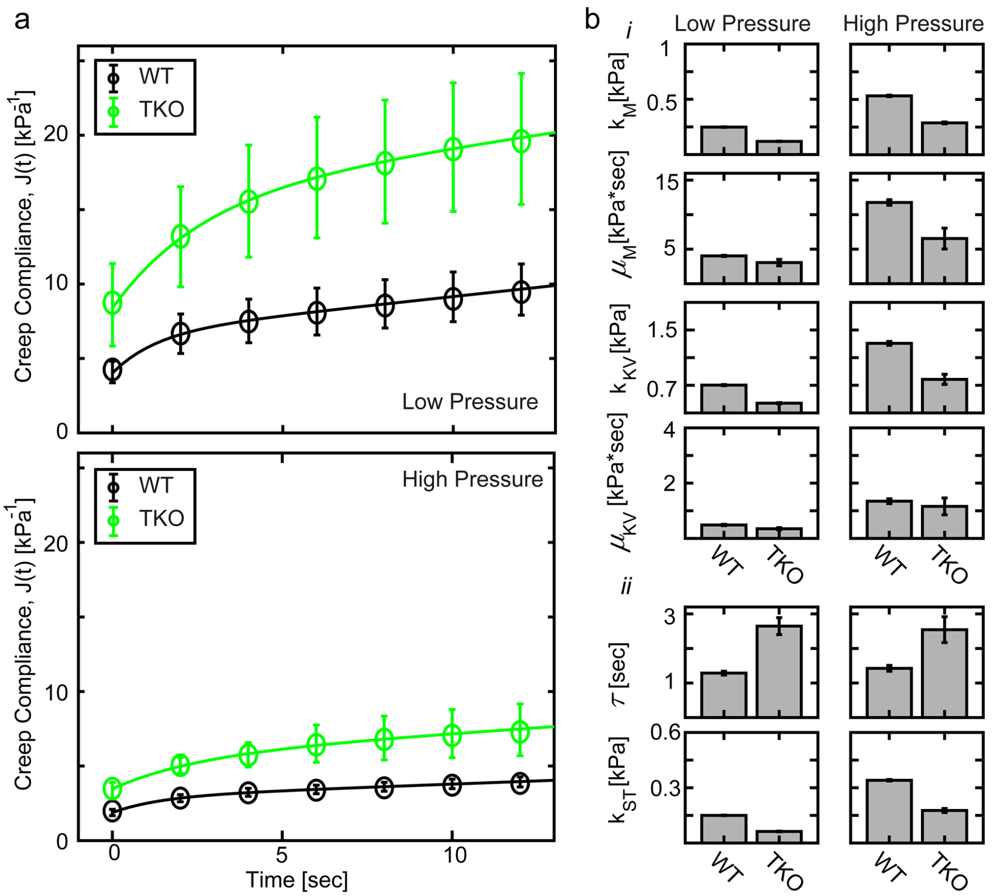

Figure S4 | Nuclear Burgers response under low and high loading levels: (a) MPA of WT and TKO nuclei was performed using low (< 0.8 kPa) and high (> 1.6 kPa) aspiration pressure. For both regimes, a Burgers response was obtained with decreased compliance at high pressure. (b) Fit estimates of (i) the viscoelastic Burgers parameters and (ii) the physical parameters τ and k_{ST} are shown for low and high loading experiments. Number of WT nuclei measured at low and high loading: $n_{Low} = 13$ and $n_{High} = 12$. Number of TKO nuclei measured at low and high loading: $n_{Low} = 10$ and $n_{High} = 7$.

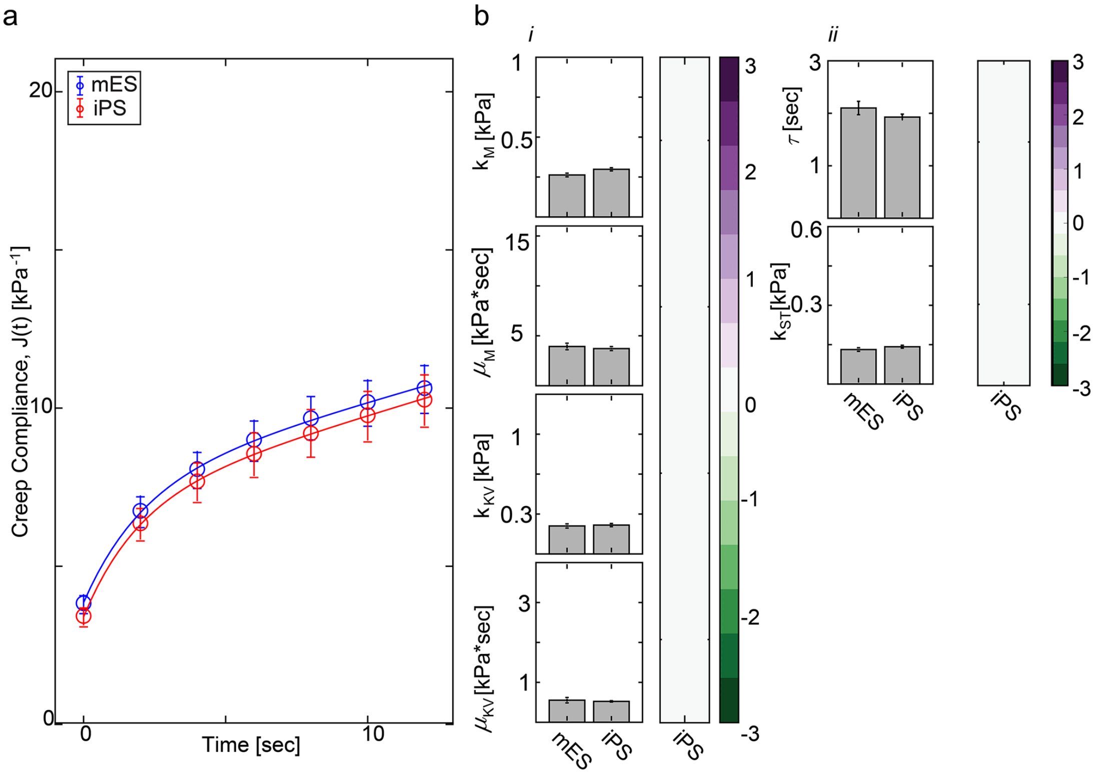

Figure S5 | Nucleus mechanics of pluripotent stem cells conforms to the Burgers model: (a) Nuclear creep compliance of mouse embryonic stem (mES) cells and induced pluripotent stem (iPS) cells, as measured by micropipette aspiration, is well fitted by the four-elements Burgers model (R-square > 0.99.). (b-i) Nucleus mechanics of mES and iPS cells is indistinguishable, as reflected by (i) their Burgers elements and (ii) their viscoelastic response time and effective steady state stiffness. Right panels show negligible log2-fold iPS/mES ratios. n > 20 cells of each cell type. Error bars are the 95% fitting confidence boundaries.

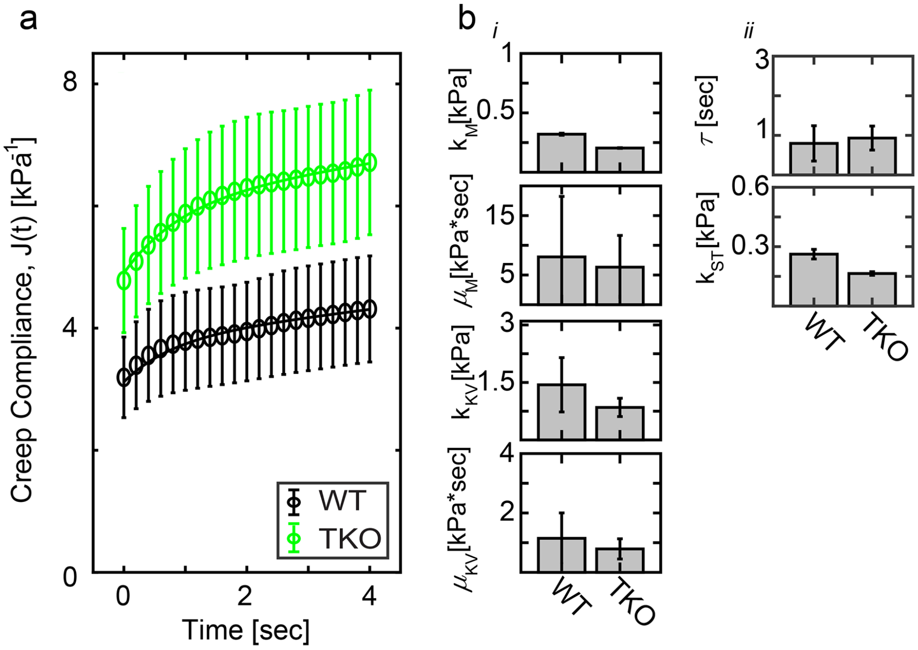

Figure S6 | Nuclear Burgers response is obtained by nano-indentation measurements: (a) Nuclear creep test of WT and TKO cells adhering to glass surface was performed by nano-indentation ($f = 1\ \mu N$). A Burgers response was obtained showing higher compliance of TKO nuclei. (b) Fit results of (i) the Burgers elements and (ii) the physical parameters τ and k_{ST}. Number of measured TKO nuclei: $n_{WT} = 6$ and $n_{TKO} = 18$. Error-bars mark 95% confidence intervals.

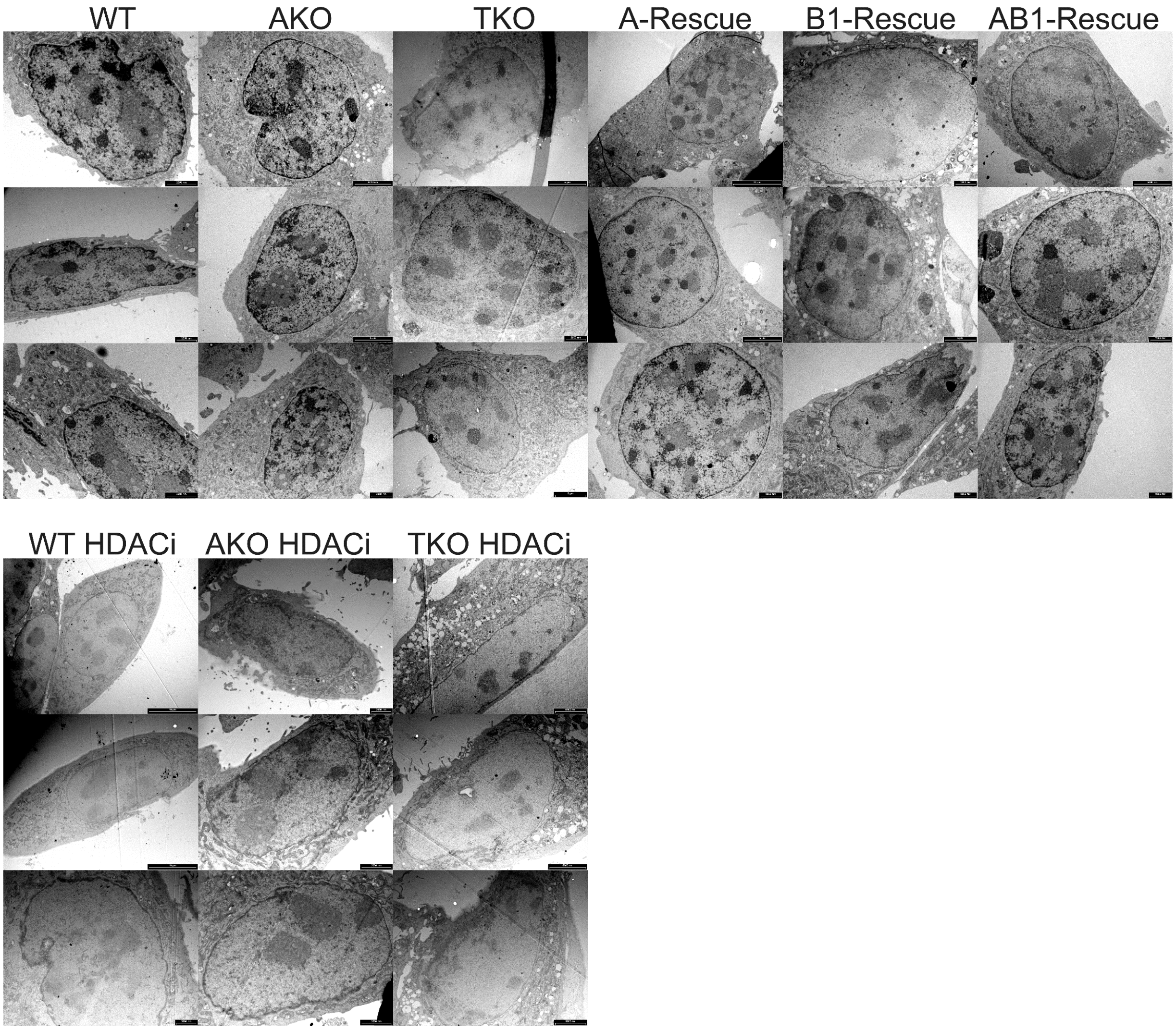

Figure S7 | Peripheral and nucleoplasmic heterochromatin condensation is regulated by lamins and histone de-acetylation. Three representative TEM images of cell nuclei stained with osmium tetroxide and potassium ferricyanide are shown for each cell line. Both peripheral and nucleoplasmic levels of condesed chromatin are high in lamin expressing cells and low in HDACi-treated cells.

Table-S1: Statistical significance of the viscoelastic differences between A- and B1-resuce nuclei.

Lamin-A Vs B1 rescue nuclei	k_M	μ_M	k_{KV}	μ_{KV}	τ	k_{ST}
*p-value	**0.46**	**0.07**	**0.10**	**0.10**	**0.02**	**0.36**

* Student's t-test p-values were calculated between the subsets of lamin-A and lamin-B1 rescue nuclei.

Table-S2: Statistical significance of the viscoelastic differences between A- and B1-resuce nuclei.

**R-square**	**Burgers**	**SLS Kelvin-representation**	**SLS Maxwell-representation**
**WT**	0.994±0.004	0.978±0.015	0.978±0.015
**AKO**	0.993±0.007	0.965±0.029	0.968±0.030
**TKO**	0.994±0.006	0.971±0.026	0.973±0.027
**LA**	0.985±0.012	0.970±0.029	0.970±0.029
**S22A**	0.990±0.008	0.970±0.031	0.970±0.031
**S22D**	0.995±0.004	0.983±0.005	0.986±0.007
**LB1**	0.977±0.027	0.896±0.060	0.896±0.060
**LAB1**	0.973±0.068	0.939±0.076	0.939±0.076
**WT-HDACi**	0.967±0.093	0.948±0.053	0.948±0.053
**AKO-HDACi**	0.985±0.019	0.916±0.053	0.924±0.044
**TKO-HDACi**	0.991±0.016	0.954±0.032	0.965±0.020
**mES**	0.989±0.019	0.936±0.055	0.950±0.039
**Models**	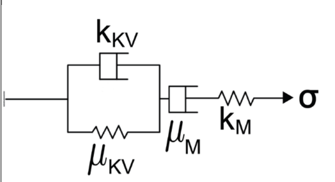	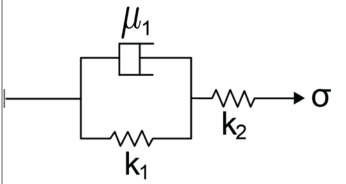	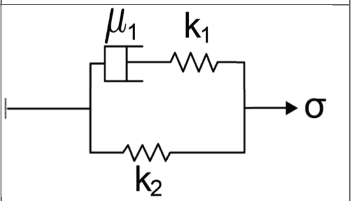
